# Antifungal activity of hypocrellin compounds and their synergistic effects with antimicrobial agents against *Candida albicans*

**DOI:** 10.1111/1751-7915.13601

**Published:** 2020-06-08

**Authors:** Shihao Song, Xiuyun Sun, Lili Meng, Qianhua Wu, Ke Wang, Yinyue Deng

**Affiliations:** ^1^ School of Pharmaceutical Sciences (Shenzhen) Sun Yat‐sen University Guangzhou 510275 China; ^2^ College of Agriculture South China Agricultural University Guangzhou 510642 China; ^3^ Pulmonary and Critical Care Medicine Ward The First Affiliated Hospital of Guangxi Medical University Nanning 530021 China

## Abstract

*Candida albicans* is a common human fungal pathogen. The previous study revealed that quinone compounds showed antimicrobial activity against *C. albicans* by inhibiting cell growth. However, it was unclear whether quinones have other antifungal effects against *C. albicans* in addition to fungicidal effects. In this study, we assessed the inhibitory activity of a total of 25 quinone compounds against *C. albicans* morphological transition, which is essential for the pathogenicity of *C. albicans*. Several quinones exhibited strong inhibition of mycelium formation by *C. albicans* SC5314. Three leading compounds, namely hypocrellins A, B and C, also exhibited marked attenuation of *C. albicans* SC5314 virulence in both human cell lines and mouse infection models. These three compounds significantly suppressed the proliferation of *C. albicans* SC5314 cells in a mouse mucosal infection model. Intriguingly, hypocrellins not only attenuated the cytotoxicity of a nystatin‐resistant *C. albicans* strain but also showed excellent synergistic effects with antifungal agents against both wild‐type *C. albicans* SC5314 and the drug‐resistant mutant strains. In addition, hypocrellins A, B and C interfered with the biological functions and virulence of various clinical *Candida* species, suggesting the promising potential of these compounds for development as new therapeutic agents against infections caused by *Candida* pathogens.

## Graphical abstract

In this study, we assessed the inhibitory activity of a total of 25 quinone compounds against *Candida albicans* morphological transition, which is essential for the pathogenicity of *C. albicans*. Three leading compounds, namely, hypocrellins A, B and C, exhibited strong inhibition of hypha formation, biofilm formation and virulence by *C. albicans* SC5314 and several other *Candida* strains. Our findings suggest the promising potential of these compounds for development as new therapeutic agents against infections caused by *Candida* pathogens.

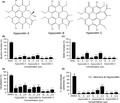

## Introduction

*Candida albicans* is one of the most common pathogenic fungi in humans, causing mild mucosal infections and high morbidity and mortality in immunocompromised individuals, especially in patients with AIDS or those treated with cancer chemotherapy or transplantation procedures (Pfaller and Diekema, 2010). In HIV/AIDS patients, at least 80% of oropharyngeal candidiasis (OPC) is caused by *C. albicans* (Sangeorzan *et al*., 1994; Revankar *et al*., 1998). It is estimated that the annual cost of treating *Candida* infections is more than $1 billion (Miller *et al*., 2001; Pappas *et al*., 2003). Long‐term clinical treatment of *C. albicans* infections with antifungal drugs has led to the development of drug resistance (Casalinuovo *et al*., 2004). Therefore, there is an urgent need for new therapeutic regimens to prevent or control diseases caused by *C. albicans* (Zhao *et al*., 2018; Meng *et al*., 2019a; Meng *et al*., 2019b).

One of the main virulence characteristics of *C. albicans* is the ability of this organisms to undergo morphological transformation between two forms: yeast and mycelium (Odds and Kerridge, 1985; Odds, 1987; Leberer *et al*., 1997; Stoldt *et al*., 1997; Liu, 2001; Zheng *et al*., 2004). The most widely studied morphological transformation, from yeast form to mycelium form, is caused by various environmental factors, including pH, nitrogen and/or carbon starvation, high temperature and serum (Ernst, 2000). This transition involves various interconnected signalling pathways, including the cAMP and MAPK pathways (Biswas *et al*., 2007). A key component of the cAMP pathway is the adenylate cyclase Cdc35, which is essential for hyphal growth (Rocha *et al*., 2001). The MAP kinase pathway and the cAMP/PKA pathway share a common upstream activator, Ras1 (Feng *et al*., 1999; Leberer *et al*., 2001; Martin *et al*., 2005).

Quinones are a class of compounds that have many biological activities, such as antibacteria, antioxidant, antitumor and anti‐HIV activities, and these biological activities are associated with the redox properties of the carbonyl functionality of these compounds (Ma *et al*., 2004; Pérez‐Sacau *et al*., 2007; Eyong *et al*., 2008; Hassan *et al*., 2016). Among these compounds, hypocrellins A, B and C are isolated from the parasitic fungus *Hypocrella bambusae* (Berk. Et Broome) Sacc. These agents have been used as traditional medicines to treat rheumatoid arthritis, gastric diseases (Su *et al*., 2011) and dermatosis caused by fungi (Wan and Chen, 1981; Wang and Bao, 1985). Previous reports have shown that hypocrellins have photodynamic anticancer (Cao and Cheng, 1988; Diwu, 1995; Miller *et al*., 1997; Zhang *et al*., 1998) and antiviral activities (Hudson *et al*., 1992; Hirayama *et al*., 1997). These activities are contributed by the ability of these compounds to produce active oxygen (Park *et al*., 1998; Ali *et al*., 2002) and inhibit protein kinase C activity (Diwu *et al*., 1994). In this study, we showed that hypocrellins A, B and C have strong inhibitory effects on the mycelium formation, biofilm formation and pathogenicity of various *Candida* species at micromolar concentrations. Additionally, we constructed a murine model of OPC and found that hypocrellins A, B and C significantly increased fungal pathogen clearance. We also demonstrated that these compounds have efficacy against a drug‐resistant *C. albicans* strain which was resistant to nystatin and exhibit favourable synergistic effects with antifungal drugs against both wild‐type and the drug‐resistant strains of *C. albicans* SC5314. Our results suggest that hypocrellins A, B and C offer a promising potential solution for the treatment of infections caused by wild‐type or drug‐resistant *Candida* strains.

## Results

### Quinone compounds inhibit mycelium formation by *C. albicans* SC5314

**Fig. 1:**
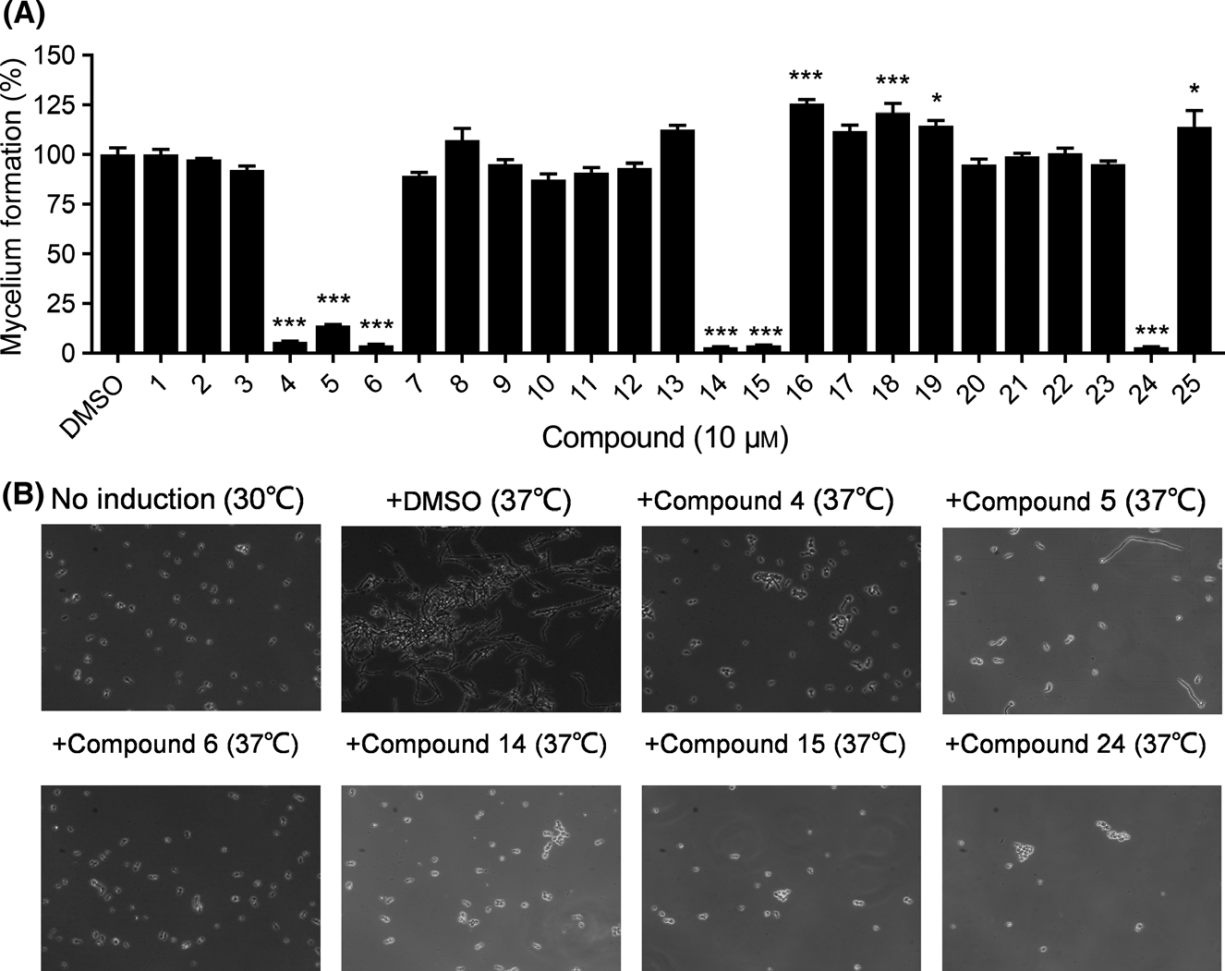
Effects of quinones on *C. albicans* SC5314 mycelium formation. A. At least six compounds (compounds **4**, **5**, **6**, **14**, **15** and **24**) reduced the mycelium formation of *C. albicans* cells by more than 85%. Data are means ± standard deviations from three independent experiments. *P < 0.05; ***P < 0.001 vs DMSO (unpaired *t*‐test). B. Images of mycelium formation by *C. albicans* SC5314 in the absence and presence of the six compounds (compounds **4**, **5**, **6**, **14**, **15** and **24**) at a final concentration of 10 μM after induction for 6 h at 37°C.

*Candida albicans* virulence is closely associated with the morphological transition between yeast and hyphae (Blankenship and Mitchell, 2006; Biswas *et al*., 2007). Therefore, under the conditions of mycelial induction at 37°C, we studied the effects of a series of quinone compounds on the transition of *C. albicans* from yeast to hyphae *in vitro*. After 6 h of induction, in the control group, most of the *C. albicans* cells had formed hyphae, while the addition of some quinone compounds inhibited mycelium formation (Fig. 1, Table S1). When the final concentration was 10 μM, at least six compounds (compounds **4** [hypocrellin A], **5** [hypocrellin B], **6** [hypocrellin C], **14** [juglone], **15** [plumbagin] and **24** [Beta‐Hydroxyisovalerylshikonin]) reduced mycelium formation by *C. albicans* cells by more than 85% (Fig. 1A). Among these compounds, the inhibition rates of compounds **6**, **14**, **15** and **24** against mycelium formation by *C. albicans* SC5314 were approximately 95% (Fig. 1A).

### Quinone compounds combat *C. albicans* SC5314 virulence

**Fig. 2:**
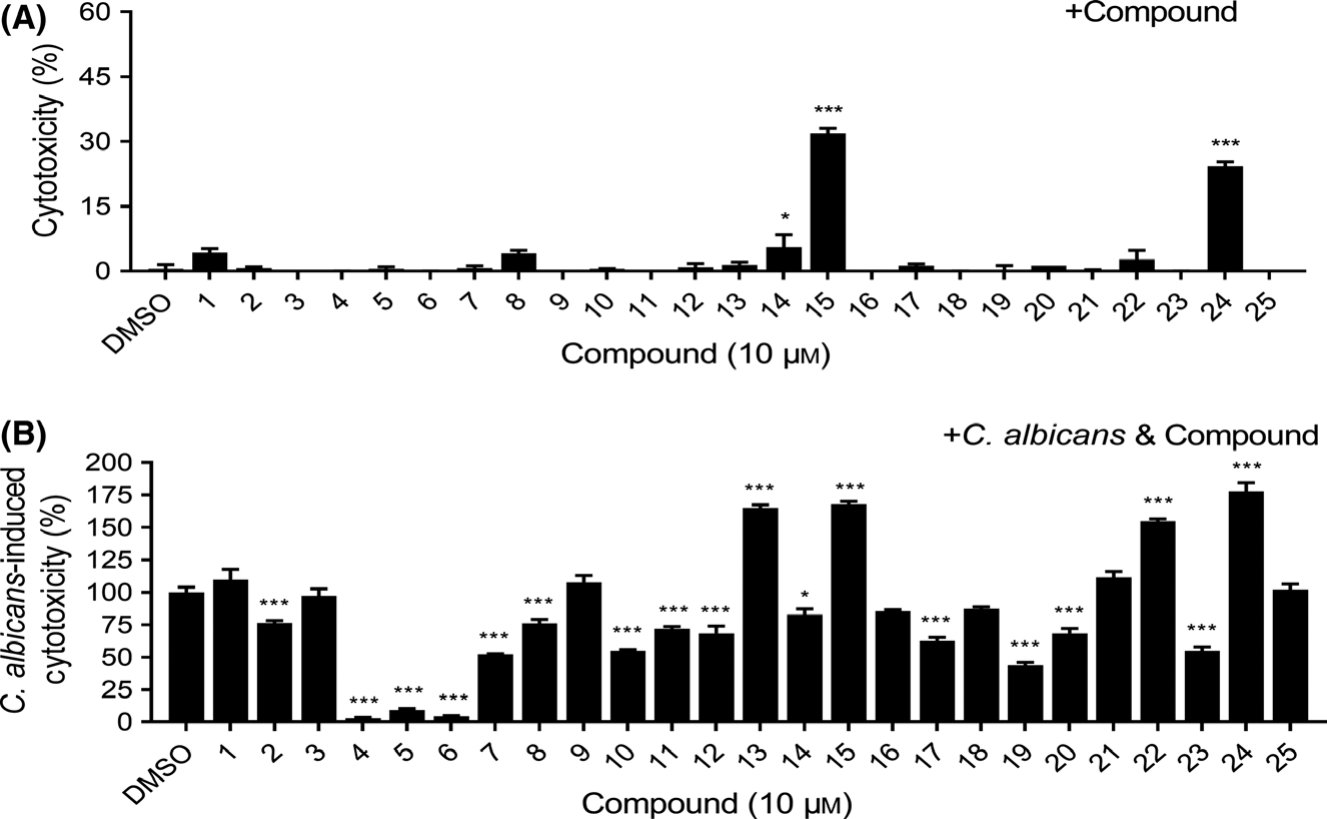
Effects of quinones on *C. albicans* SC5314 virulence. A. Analysis of the toxicity of the quinones against A549 cells. B. Analysis of the effects of the quinones on the cytotoxicity of *C. albicans* against A549 cells. Cytotoxicity detected by LDH release. Data are means ± standard deviations from three independent experiments. *P < 0.05; ***P < 0.001 vs DMSO (unpaired *t*‐test).

**Fig. 3:**
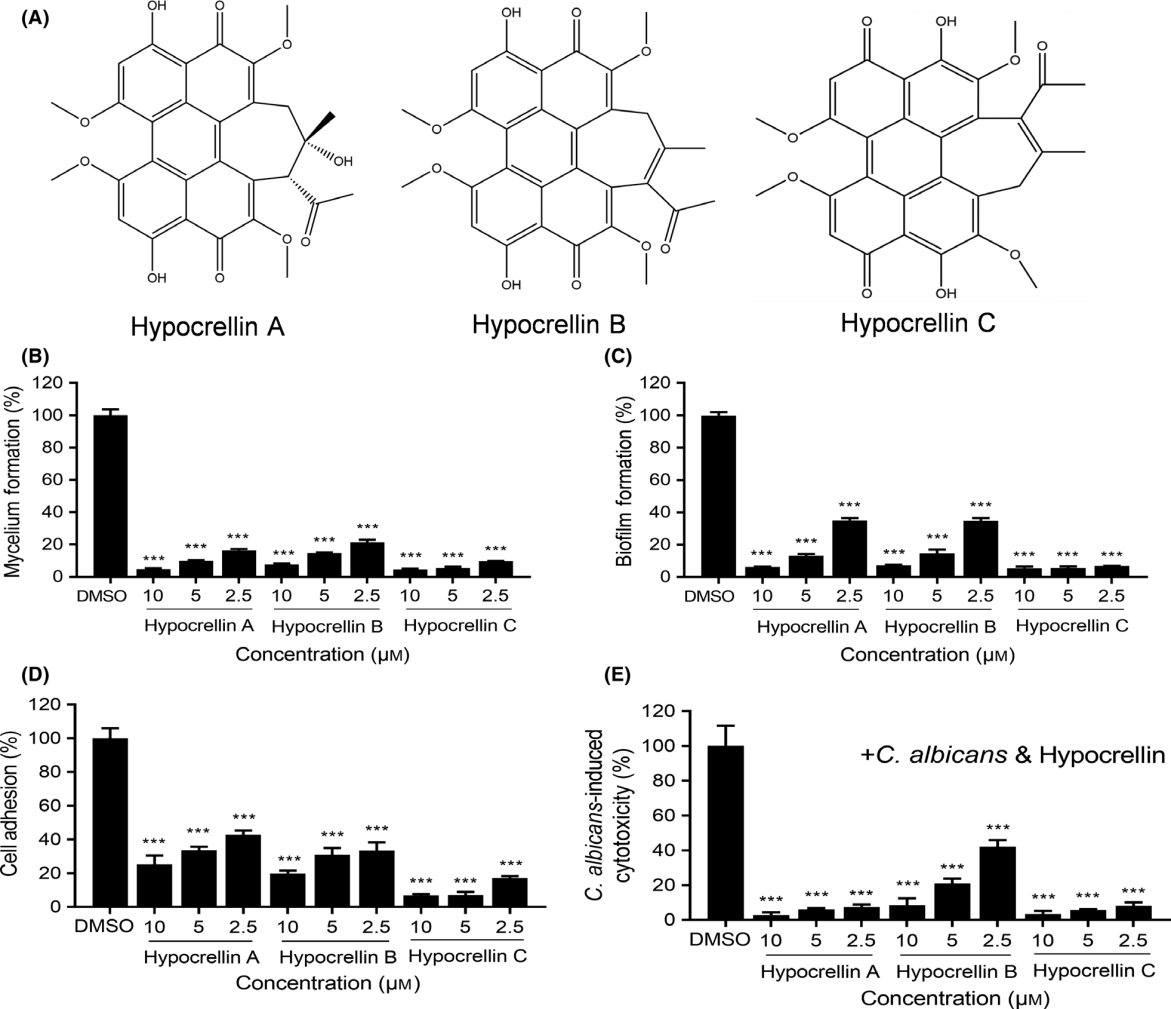
Chemical structures of hypocrellins A, B and C (A), and effects of different concentrations of hypocrellins on *C. albicans* SC5314 mycelium formation (B), biofilm formation (C), cell adhesion (D) and *C. albicans*‐induced cytotoxicity (E). Data are means ± standard deviations from three independent experiments. ***P < 0.001 vs DMSO (unpaired *t*‐test).

We found that at least six quinone compounds reduced mycelium formation by *C. albicans* SC5314, so we studied whether the quinone compounds could reduce the virulence of *C. albicans* SC5314 against human cells. At a final concentration of 10 μM, most of quinones showed no cytotoxicity against A549 human cells (Fig. 2A), excluding compound **15** (plumbagin), compound **24** (Beta‐Hydroxyisovalerylshikonin), compound **14** (juglone), compound **1** (alkannin) and compound **8** (1,3,6‐Tri‐O‐galloyl‐beta‐D‐glucose). Interestingly, we found that exogenous addition of quinone compounds reduced the cytotoxicity of *C. albicans* SC5314 (Fig. 2B). Among these compounds, compounds **4**, **5** and **6** inhibited the cytotoxicity of *C. albicans* SC5314 by more than 90% at a final concentration of 10 μM (Fig. 2B). Because compounds **4**, **5** and **6** (hypocrellins A, B and C, respectively, Fig. 3A) also effectively inhibited mycelium formation by *C. albicans* SC5314 (Fig. 1), these compounds were selected for further study.

### Hypocrellins are only slightly toxic to *C. albicans* SC5314 cells

At a final concentration of 10 µM, hypocrellins A, B and C (Fig. 1; see also Table S1) considerably reduced *C. albicans* mycelium formation by more than 85% but only slightly inhibited the growth of *C. albicans* SC5314 under these conditions (Fig. S1A). The used concentration of these compounds was also much lower than their MICs against *C. albicans* SC5314 (Table 1). This result was further supported by Evan’s blue staining experiment, and we found that the mortalities of *C. albicans* cells treated by hypocrellins at a final concentration of 10 µM were only 10.69%, 8.71% and 6.63%, respectively (Fig. S1B and C). Then, we chose this concentration and continued to study the effects of hypocrellins on *C. albicans*.

**Table 1:** Minimum inhibitory concentrations (MICs) of the hypocrellin compounds and antifungal agents against the *Candida* strains used in this study.

Strains	Fluconazole MIC (μg ml^−1^)		Nystatin MIC (μg ml^−1^)	Hypocrellin A MIC (μM)	Hypocrellin B MIC (μM)	Hypocrellin C MIC (μM)
*C. albicans* SC5314	4	5		80	> 100	> 100
*C. glabrata* ATCC 2001	4	2.5		> 100	> 100	> 100
*C. albicans* ATCC 10231	4	5		40	> 100	> 100
*C. albicans* ATCC 90028	0.5	5		80	> 100	> 100
*C. tropicalis* ATCC 750	1	5		> 100	> 100	> 100
*C. albicans* ATCC 14053	< 0.5	2.5		> 100	> 100	> 100
*C. albicans* Nystatin‐resistant strain	4	100		80	> 100	> 100

### Hypocrellins inhibit mycelium formation, biofilm formation, cell adhesion and virulence of *C. albicans* SC5314 in a dose‐dependent manner

To determine whether the effects of hypocrellins A, B and C on *C. albicans* SC5314 are dose dependent, we first studied the effects of different concentrations of these hypocrellin compounds on mycelium formation (Fig. 3B). The results showed that all three hypocrellins exhibited dose‐dependent activity. They all inhibited *C. albicans* mycelium formation by more than 70% at a final concentration of 2.5 µM (Fig. 3B).

*Candida albicans* biofilms that form on abiotic surfaces, including catheters and dentures, or on biotic surfaces, including typical mucosal cell surfaces, are important virulence factors in human pathology. We therefore studied the inhibitory effects of hypocrellins on *C. albicans* SC5314 biofilm formation and cell adhesion to polystyrene and human cells respectively. Interestingly, hypocrellins A, B and C reduced the biofilm formation of *C. albicans* by approximately 90% at a final concentration of 10 µM (Fig. 3C). Different concentrations of hypocrellins A, B and C, from 2.5 to 10 µM, were tested for inhibition of *C. albicans* biofilm formation in a dose‐dependent manner (Fig. 3C).

Hypocrellins A, B and C also reduced the cell adhesion of *C. albicans* to A549 cells to approximately 25.2%, 19.8% and 6.9% at a final concentration of 10 µM (Fig. 3D). The inhibitory effects of different concentrations of hypocrellins A, B and C, from 2.5 to 10 µM, on *C. albicans* cell adhesion to A549 cells were also determined to be dose dependent (Fig. 3D).

Moreover, exogenous addition of hypocrellins caused significant inhibition of the cytotoxicity of *C. albicans* SC5314 against A549 cells (Fig. 3E). When the final concentration of hypocrellins A, B and C was 10 µM, the inhibition rates of hypocrellins against *C. albicans* cytotoxicity were as high as 90% or more (Fig. 3E). Hypocrellins A and C exhibited strong protection to A549 cells even at a low concentration of 2.5 µM (Fig. 3E). We then continued to analyse the cytotoxicity of hypocrellin compounds on human A549 cells and normal human intestinal epithelial cells (HIEC) and test the inhibitory activities of these compounds against *C. albicans* cytotoxicity on the two cell lines by using MTT assay (Fig. S2). The results showed that hypocrellins had only weak toxicity on A549 cells (Fig. S2A) and no toxicity on HIEC cells (Fig. S2C) at a final concentration of 10 µM. These compounds also exhibited an obvious inhibition against *C. albicans* cytotoxicity on both A549 cells (Fig. S2B) and HIEC cells (Fig. S2D) at the indicated concentrations.

### Hypocrellins attenuate *C. albicans* SC5314 pathogenicity in mouse infection models

**Fig. 4:**
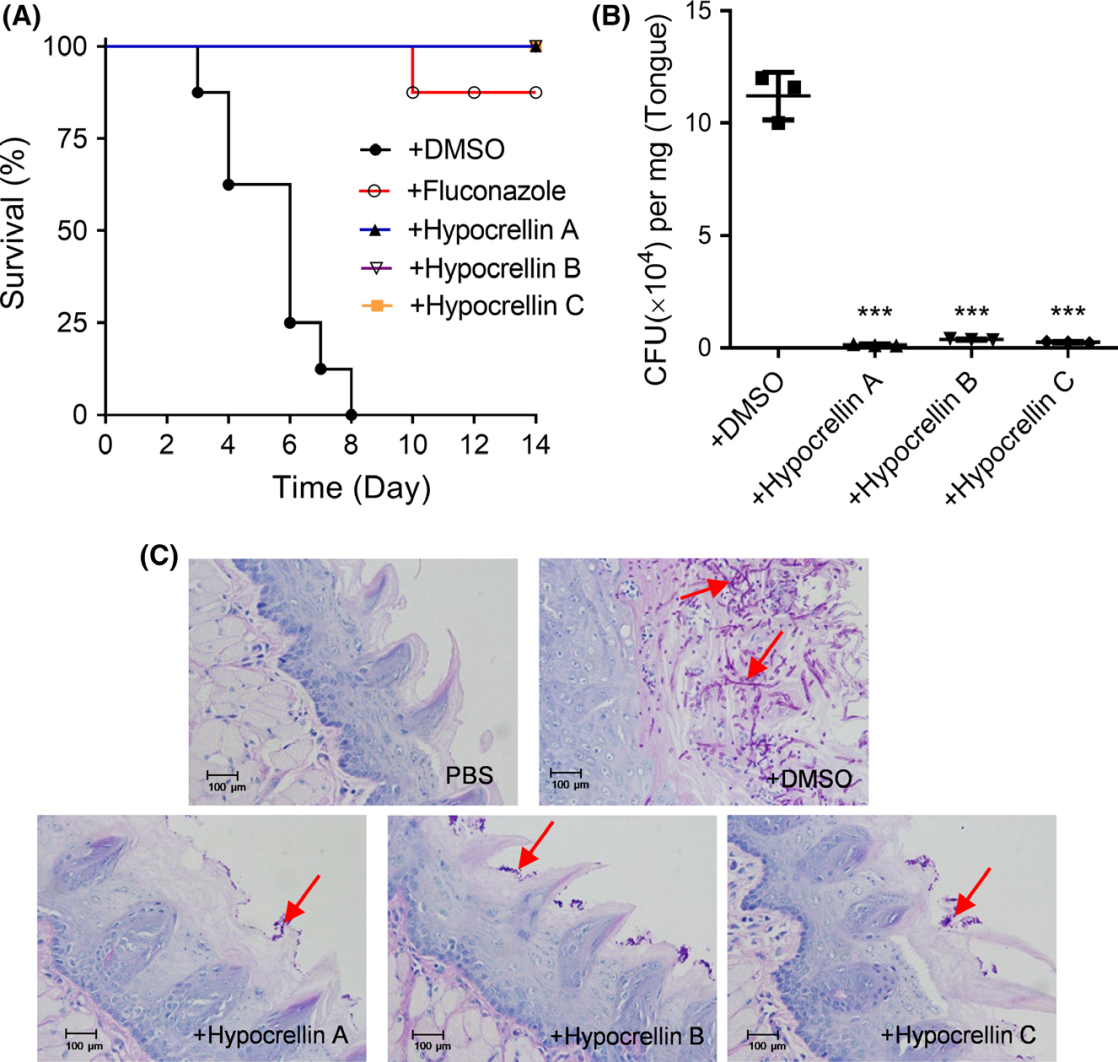
Efficacy of hypocrellins A, B and C (10 μM) against *C. albicans* SC5314 in the mouse infection models. A. Survival rates of mice after infection with *C. albicans* SC5314 with and without the treatment of hypocrellins A, B and C. B. The *in vivo* pathogen cell numbers of *C. albicans* in mouse tongue after infection with *C. albicans* SC5314 with and without the treatment of hypocrellins A, B and C. C. Pathological sections of the mouse tongues after infection with *C. albicans* SC5314 with and without the treatment of hypocrellins A, B and C. The red arrows showed the infection sites in the mouse tongues by *C. albicans* SC5314. Data are means ± standard deviations from three independent experiments. ***P < 0.001 vs *C. albicans* + DMSO (unpaired *t*‐test).

*Candida albicans* is a common apathogenic species, which may lead to life‐threatening systemic infection in people with low immunity (Sangeorzan *et al*., 1994; Rhodus *et al*., 1997; Willis *et al*., 1999). Due to the excellent activity of hypocrellins in the reduction of biofilm formation, mycelium formation and cytotoxicity of *C. albicans* SC5314 cells, we further investigated whether hypocrellins attenuated *C. albicans* SC5314 pathogenicity in mouse infection models, including in the systemic infection model and the OPC infection model. The mouse model assays demonstrated similar results as the cellular model. Addition of hypocrellins led to a clear reduction in the virulence of *C. albicans* SC5314 in the mouse infection models (Fig. 4). At 10 d postinfection, the mortality of mice infected with *C. albicans* alone was 100%, while the mortalities of mice infected with *C. albicans* and treated with fluconazole and hypocrellins A, B and C were 12.5%, 0, 0 and 0 respectively (Fig. 4A).

Further investigation was conducted to analyse the symptoms of mouse tongues infected by *C. albicans* SC5314 because the tongue is one of the most important niches for *C. albicans* cells. In the treatment groups of hypocrellins, the level of *C. albicans* infection in the mouse tongue decreased significantly (Fig. 4B), and pathological sections of the mouse tongue also showed that there was almost no *C. albicans* cells after treatment with hypocrellins (Fig. 4C).

### Hypocrellins inhibit *C. albicans* SC5314 mycelium formation mostly by interfering with the cAMP‐PKA and MAPK pathways

**Fig. 5:**
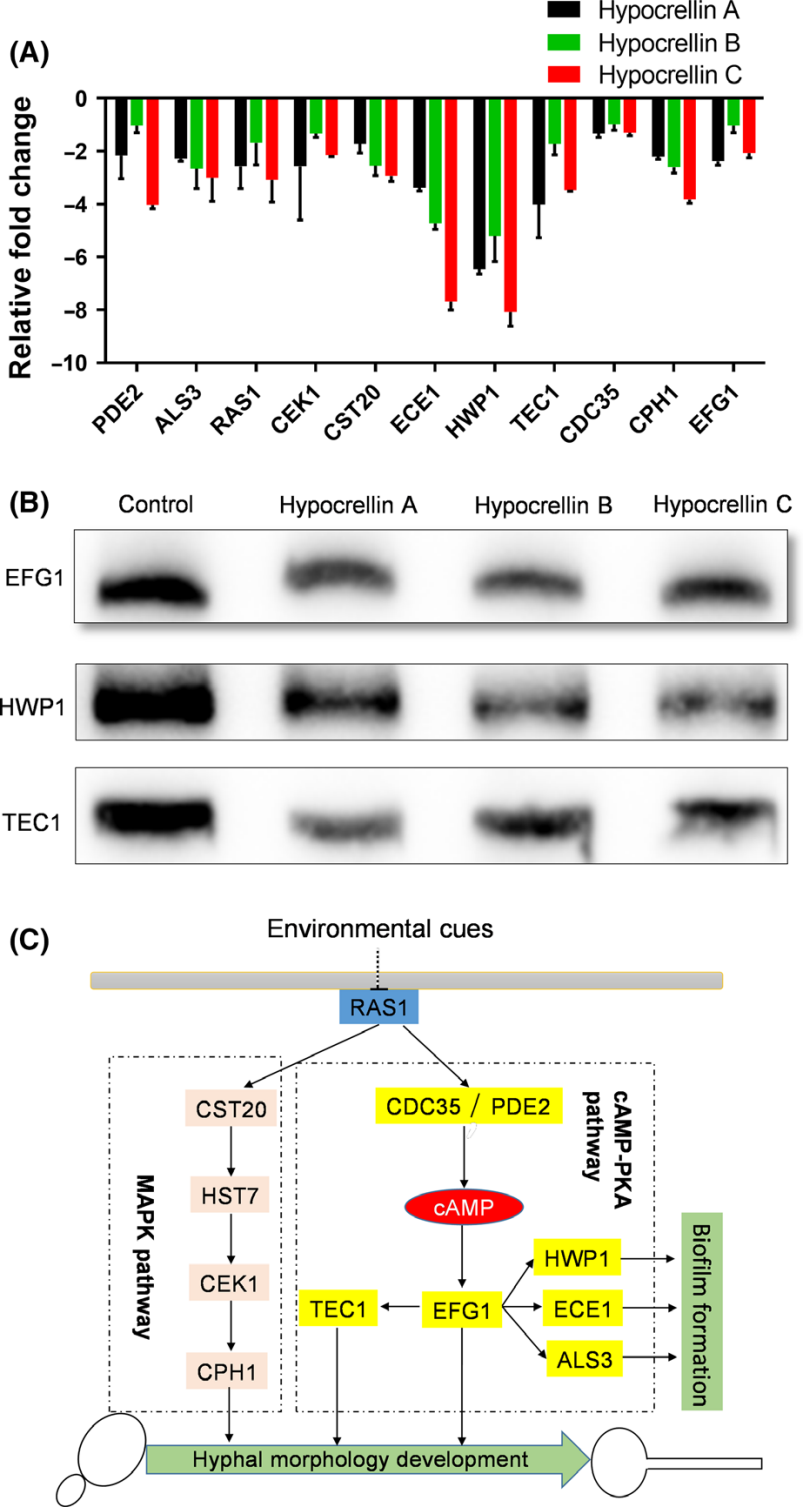
Effects of hypocrellins A, B and C (10 μM) on the signalling pathways involved in the mycelium development process. A. Comparison of relative fold changes of regulator‐encoding genes between *C. albicans* cells with and without the addition of hypocrellins A, B and C. B. Western blotting analysis of the amounts of EFG1, HWP1 and TEC1 in *C. albicans* cells with and without the treatment of hypocrellins A, B and C. C. Schematic diagram of the signalling pathways that govern mycelium morphogenesis in *C. albicans* affected by hypocrellins A, B and C.

To study the mechanism by which hypocrellins inhibit mycelium formation by *C. albicans*, we further studied whether hypocrellins interfered with the signalling pathways associated with mycelial development (Sudbery *et al*., 2004; Lu *et al*., 2011; Shareck and Belhumeur, 2011; Hnisz *et al*., 2012). *C. albicans* mycelium formation is mostly associated with two known signalling pathways: the MAPK and cAMP‐PKA pathways. We analysed the effects of hypocrellins on mycelium‐specific genes using quantitative real‐time PCR. The results demonstrated that hypocrellins clearly decreased the expression levels of relevant genes in both the cAMP‐PKA (PDE2, CDC35, EFG1, TEC1, HWP1, ECE1, ALS3) and MAPK (CST20, CEK1, CPH1) pathways (Fig. 5A). We then further used Western blotting assay to analyse the effects of hypocrellins on the two pathways. It was shown that addition of hypocrellins significantly inhibited the amounts of EFG1, HWP1 and TEC1 proteins (Fig. 5B). As EFG1 and TEC1 promote mycelium formation, and EFG1 and HWP1 control biofilm formation in *C. albicans*, so hypocrellins disrupt the mycelium formation and biofilm formation of *C. albicans* mostly by interfering with the cAMP‐PKA and MAPK pathways (Fig. 5C).

### Hypocrellins inhibit the biological functions and pathogenicity in various clinical *Candida* isolates

**Fig. 6:**
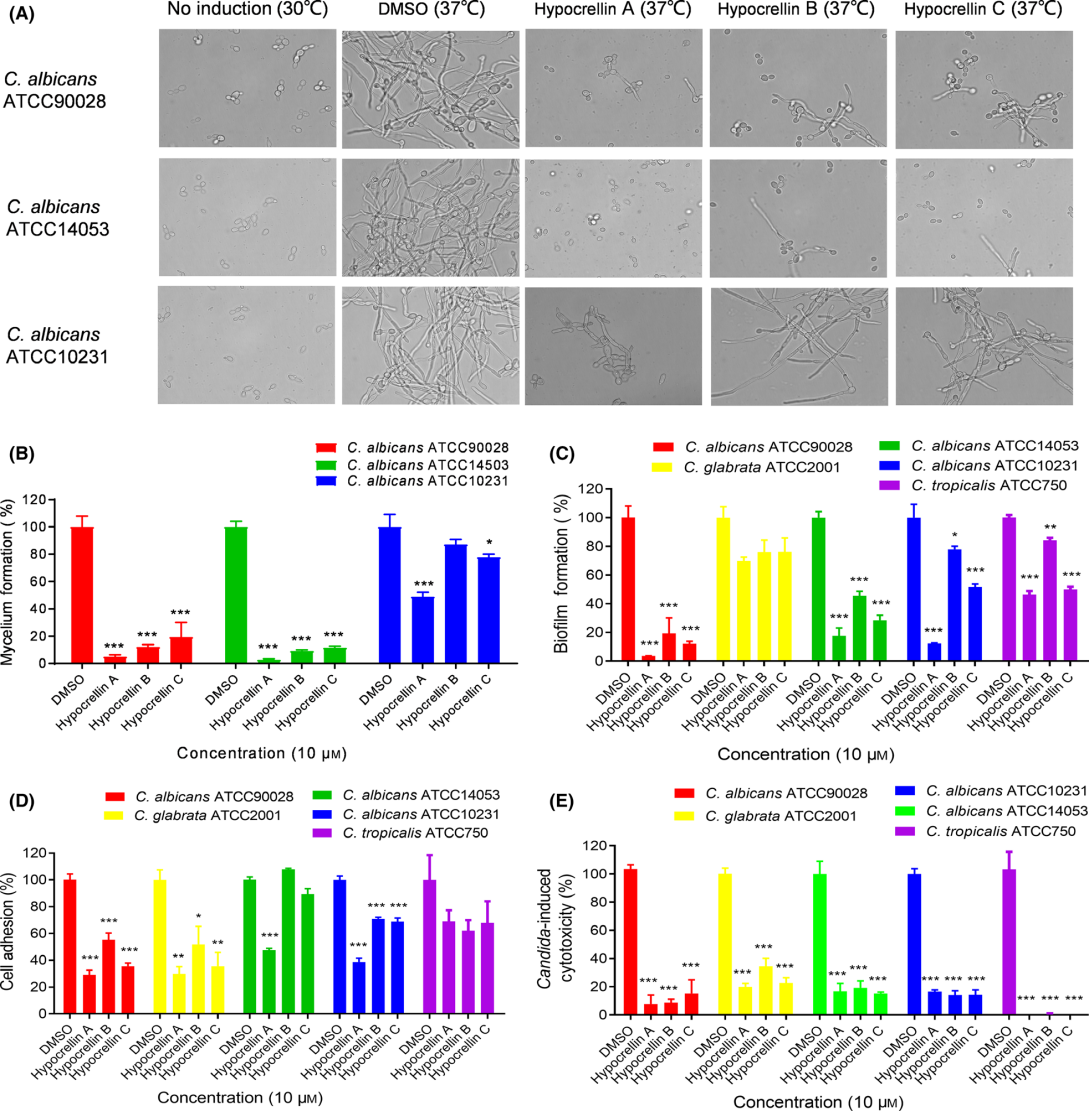
Influence of hypocrellins A, B and C (10 μM) on mycelium formation, biofilm formation, cell adhesion and virulence of various *Candida* isolates. (A) Images of mycelium formation by clinical *C. albicans* strains in the absence and presence of hypocrellins A, B and C after induction for 6 h at 37°C. Effects of hypocrellins A, B and C on mycelium formation (B), biofilm formation (C), cell adhesion (D) and virulence (E) of *Candida* isolates. Data are means ± standard deviations from three independent experiments. *P < 0.05; **P < 0.01; ***P < 0.001 vs DMSO (unpaired *t*‐test).

To study whether hypocrellins have conserved effects on *Candida* species, we chose two more different *Candida* species (*C. glabrata* and *C. tropicalis*) and several *C. albicans* strains and studied the effects of hypocrellins on the mycelium formation, biofilm formation, cell adhesion and virulence of these strains. Intriguingly, hypocrellins exhibited strong inhibition of mycelium formation in *C. albicans* ATCC 90028 and ATCC 14503, while showed a modest effect in *C. albicans* ATCC 10231 (Fig. 6A and B). Hypocrellins A, B and C strongly inhibited biofilm formation by *C. albicans* ATCC 90028 and ATCC 14053 strains (Fig. 6C), and partially reduced the adhesion of these *Candida* strains (Fig. 6D). More interesting, addition of hypocrellins A, B and C at 10 µM strongly inhibited the cytotoxicity of all these tested strains including *C. albicans* ATCC 90028*, C. albicans* ATCC 14053*, C. albicans* ATCC 10231, *C. glabrata* ATCC 2001 and *C. tropicalis* ATCC 750 against A549 cells (Fig. 6E).

### Combination of hypocrellin compounds with nystatin synergistically decreases the virulence of a nystatin‐resistant *C. albicans* mutant strain *in vitro*

**Fig. 7:**
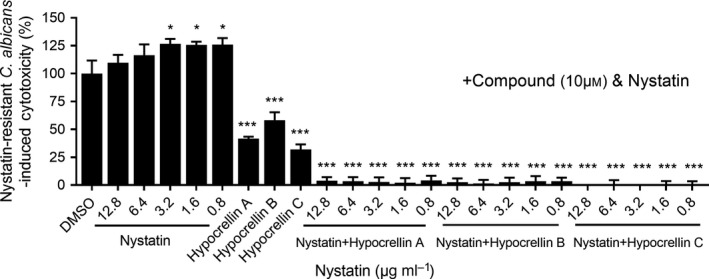
Effects of hypocrellins A, B and C and their synergistic activities with nystatin on the virulence of a nystatin‐resistant *C. albicans* SC5314 mutant strain. Data are means ± standard deviations from three independent experiments. *P < 0.05; ***P < 0.001 vs DMSO (unpaired *t*‐test).

Synergistic action of antimicrobial agents for improved efficacy or to overcome bacterial or fungal resistance has become a common treatment method. As hypocrellin compounds showed excellent activity against the biological functions and virulence of *C. albicans*, we continued to investigate whether hypocrellin compounds have synergistic activity with antifungal agents against drug‐resistant *C. albicans* strains in addition to antifungal activities. Nystatin is a common drug for the treatment of fungal infections in the mouth, stomach and intestines. This drug inhibits fungal growth and prevents infections. The results showed that exogenous addition of nystatin at 12.8 μg ml^−1^ did not inhibit the cytotoxicity of a nystatin‐resistant *C. albicans* strain (Fig. 7). Intriguingly, addition of hypocrellin compounds can distinctly decrease the cytotoxicity of the nystatin‐resistant *C. albicans* strain; the cytotoxicity of the nystatin‐resistant *C. albicans* strain was reduced to 41.69%, 58.24% and 31.91% upon supplementation with hypocrellins A, B and C at 10 μM respectively (Fig. 7). Interestingly, the combination of hypocrellin A, B or C with nystatin showed stronger inhibition on the cytotoxicity of the nystatin‐resistant *C. albicans* strain than the addition of hypocrellin A, B or C alone. As shown in Fig. 7, the cytotoxicity of the nystatin‐resistant *C. albicans* strain against A549 cells reduced to less than 5% upon treatment with 0.8, 1.6, 3.2, 6.4 and 12.8 μg ml^−1^ nystatin in combination with hypocrellin A, B or C at 10 μM. These results demonstrated not only the synergistic effect of hypocrellin compounds with nystatin against the nystatin‐resistant *C. albicans* strain but also the potential applications of hypocrellin compounds as antifungal agents and excellent adjuvants of antifungal agents.

### Combinatorial effects of hypocrellin compounds with antifungal agents against the *C. albicans* SC5314 wild‐type strain

We then investigated the possibility of using hypocrellin compounds as adjuvants of antifungal agents against the *C. albicans* SC5314 wild‐type strain. Our results showed that hypocrellins exhibit strong synergistic activities with antifungal agents including fluconazole, nystatin, amphotericin B and voriconazole against the *C. albicans* SC5314 wild‐type strain (Table 2). In particular, the addition of hypocrellins A, B and C decreased the MIC of fluconazole against *C. albicans* SC5314 from 4 μg ml^−1^ to 0.25, 0.5 and 0.25 μg ml^−1^, representing 16‐, 8‐, and 16‐fold decline respectively (Table 2). Similarly, the addition of hypocrellins A, B and C to a *C. albicans* SC5314 culture also enhanced the fungal susceptibility to nystatin from 5 μg ml^−1^ to 0.625 μg ml^−1^, representing eightfold decline respectively (Table 2). It was revealed that the addition of hypocrellins A, B and C also have the similar synergistic effects with both amphotericin B and voriconazole to decrease their MIC values against *C. albicans* SC5314 by more than eightfold and 16‐fold respectively (Table 2).

**Table 2:** Synergistic activities of hypocrellins A, B and C at 10 μM with fluconazole, nystatin, amphotericin B and voriconazole against the *C. albicans* SC5314 wild‐type strain.

Compound (10 μM)	Fluconazole MIC (μg ml^−1^)	Nystatin MIC (μg ml^−1^)	Amphotericin B MIC (μg ml^−1^)	Voriconazole MIC (μg ml^−1^)
None	4	5	0.5	0.25
Hypocrellin A	0.25	0.625	0.0625	0.0156
Hypocrellin B	0.5	0.625	0.125	0.0156
Hypocrellin C	0.25	0.625	0.0625	0.0156

## Discussion

The first step of *C. albicans* infection is adhesion to host cells, followed by mycelial growth and biofilm formation (Alexander and Perfect, 1997; Biswas *et al*., 2007; Ferreira *et al*., 2013; Dos *et al*., 2014; Ruhnke, 2014). The yeast‐to‐mycelium transformation is an essential step in *C. albicans* infection (Braun and Johnson, 2000; Saville *et al*., 2003; Zheng *et al*., 2003). Our previous studies have identified that some compounds exhibit strong activity to inhibit mycelium formation by *C. albicans* cells (Zhao *et al*., 2018; Meng *et al*., 2019a; Meng *et al*., 2019b). The results in this study suggested that hypocrellins also showed strong inhibitory activity against mycelium formation by *C. albicans* SC5314 cells (Figs 1 and 3). Intriguingly, hypocrellins exhibited good effects against biofilm formation, adhesion and mycelium formation by various *Candida* species (Fig. 6). Biofilm formation is one of the main reasons underlying the resistance of *C. albicans* to traditional antifungal drugs. Our results suggested that hypocrellins may affect antifungal agent susceptibility via a variety of pathways, including the regulation of biofilm formation, mycelium formation, drug resistance and fungal persistence (Figs 3 and 5, Table 2). Moreover, hypocrellins also attenuated the pathogenicity of *C. albicans* SC5314 in mouse infection models (Fig. 4). In general, our study proved for the first time that hypocrellins could be designed as antivirulence agents against *C. albicans* SC5314.

Currently, the clinical treatments for candidiasis are almost totally dependent on a limited number of traditional antifungal drugs, such as nystatin, which normally kill pathogens directly (Odds *et al*., 2003; Pierce *et al*., 2013). The limitations of drug development undermine the strategies currently used in clinical treatment. Our findings suggest that hypocrellins have strong synergistic effects with antifungal agents including fluconazole, nystatin, amphotericin B and voriconazole against the *C. albicans* wild‐type strain (Table 2). One reason for the synergistic effect of hypocrellins and antibiotics may be due to the fact that hypocrellins can inhibit the biofilm formation and morphological transition of *C. albicans*, thus increase the sensitivity of *C. albicans* cells to antifungal drugs (Shanmughapriya *et al*., 2014). These compounds can also decrease the cytotoxicity of a nystatin‐resistant *C. albicans* strain and have a synergistic effect with nystatin to decrease the cytotoxicity of the nystatin‐resistant *C. albicans* strain against human cells (Fig. 7), suggesting that hypocrellins could be potentially developed as new antimicrobial agents or adjuvants of antimicrobial agents to treat the infections caused by both wild‐type and drug‐resistant *Candida* strains. Therefore, hypocrellin compounds could be developed as novel drugs to overcome the spread of drug resistance.

Hypocrellins, including hypocrellin A, hypocrellin B and hypocrellin C (Fig. 3A), are photosensitizers that were originally isolated from wild *Hypocrella bambusae* but were recently synthesized chemically or biologically (Cai *et al*., 2010; O'Brien *et al*., 2010) and exhibit most of the described advantages (Wang *et al*., 1999; An *et al*., 2003). However, low absorption during the phototherapeutic window (600–900 nm) is a serious drawback for photodynamic therapy (PDT) of solid tumours (Beck *et al*., 2003; Xu *et al*., 2003). The triplet state of hypocrellins reacts through one or both of two reactions called the type Ⅰ and type Ⅱ reactions, thereby generating reactive oxygen species (ROS), such as superoxides, hydroxyl radicals and singlet oxygen, which may cause damage to the integrity of cell membranes of microorganisms by increasing reactive oxygen species levels (Su *et al*., 2011). However, in this study, we report new biological functions of hypocrellins against mycelium formation, biofilm formation, cell adhesion and virulence in not only *C. albicans* SC5314 but also various other *Candida* species and *C. albicans* strains (Figs 3 and 6). Our findings in this study are some distinguished from the previous results, which indicated that hypocrellins could be used as fungicidal agents to direct kill the cells of *Candida* species (Ma *et al*., 2004). The difference in the MICs values of hypocrellins against *Candida* species cells may be due to the different strains used in our study from the previous study. However, these compounds showed no significant inhibitory effects on the growth of the cells of these isolates used in our study (Figs S1 and S3). Taken together, our results identified new functions of hypocrellins as antivirulence agents or adjuvants of antimicrobial agents, focusing on functional inhibition rather than killing of the pathogenic fungus, which could be used as a new method to prevent infections caused by both wild‐type strains and drug‐resistant *Candida* strains to avoid the occurrence of drug resistance.

## Experimental procedures

### Strains, culture and agents

The *Candida* strains used in this study are *C. albicans* SC5314 (ATCC MYA‐2876TM) and the nystatin‐resistant mutant strain, *C. albicans* ATCC 10231, *C. albicans* ATCC 90028, *C. albicans* ATCC 14053, *C. tropicalis* ATCC 750 and *C. glabrata* ATCC 2001 (Table 1). These fungal cells were grown in either 6.7 g l^−1^ yeast nitrogen broth lacking amino acids (YNB) supplemented with 2% glucose, YPD medium (1% yeast extract, 2% peptone and 2% dextrose) or Sabouraud medium (SD medium containing 4% maltose and 1% peptone) at 30°C with shaking at 220 r.p.m. Chemicals were purchased from Chengdu Biopurify Phytochemicals Ltd. Human lung epithelial A549 cells and normal human intestinal epithelial cells (HIEC) were incubated in Dulbecco’s modified Eagle’s medium (DMEM) containing 10% fetal bovine serum (FBS) at 37°C in 95% air/5% CO_2_.

### Screening of the drug‐resistant *C. albicans* SC5314 mutant strain

Development of resistance of *C. albicans* SC5314 to nystatin was gradually improved by adding more nystatin into the SD medium (Barchiesi *et al*., 2000). *C. albicans* cells cultured overnight were diluted in SD medium and inoculated into fresh medium to an OD_600_ of 0.1 in the presence of 0.2, 0.4, 0.8, 1.6, 3.2, 6.4, 12.8 or 25.6 μg ml^−1^ nystatin. The cells were grown at 30°C in a shaking incubator, diluted in SD medium and inoculated into fresh medium containing the same concentration of nystatin to an OD_600_ of 0.1. Then, the cells were cultured to an OD_600_ of approximately 3.0 and carried into the next cycle. A 0.5 ml aliquot of the suspension collected from each cycle was mixed with 0.5 ml of 60% glycerol and stored at −80°C until antifungal susceptibility testing as described below.

### Filamentation assays

*Candida albicans* cells were cultured overnight and washed in phosphate‐buffered saline (PBS) and then diluted in fresh YNB + 2% glucose to an optical density of 0.1 to induce hyphal growth (Lee *et al*., 1975; Gimeno *et al*., 1992; Maidan *et al*., 2005). Different concentrations of compounds were added at the final concentrations indicated, the same volume of 0.2% DMSO as the control, and the *C. albicans* cells were incubated for 6 h at 37°C. Images of cells were captured by a Leica inverted fluorescence microscope with 40× and 100× lenses.

### Biofilm formation assays

Biofilm biomass was measured directly using the crystal violet (CV) staining method (Reynolds and Fink, 2001). Overnight cultured cells of *C. albicans* SC5314 and other strains were diluted in fresh YNB + 2% glucose media to an OD_600_ of 0.1 with or without the addition of different compounds as indicated, the same volume of 0.2% DMSO as the control. The fungal cells were grown in each well of a 96‐well polystyrene plate at 37°C for 8 h without shaking. Then, the supernatants were removed, and the biofilms were washed three times with sterile PBS to remove non‐adherent cells. Next, the biofilms were stained by addition of 0.02% CV for 45 min. The plate was rinsed six to eight times with ice‐cold distilled water, and 200 µl of destaining solution (75% ethanol) was added. Quantification of CV was performed by measuring the absorbance at 590 nm using a microplate reader.

### Adhesion assays

The adhesion assay was performed as described previously (Krasowska *et al*., 2009). Briefly, A549 cells in DMEM supplemented with 10% FBS were seeded in 96‐well tissue culture plates at 0.5 × 10^3^ cells/well one night in advance and incubated at 37°C with 5% CO_2_. Overnight cultures of *C. albicans* SC5314 and other strains were diluted in fresh DMEM with 1% FBS to an OD_600_ of 0.5 with or without compounds at different concentrations as indicated, the same volume of 0.2% DMSO as the control. After incubation for 1.5 h at 37°C, the supernatants were removed, and the adherent cells were washed three times with sterile PBS to remove non‐adherent cells. Next, the adherent cells were stained with 0.02% CV for 45 min. The plate was rinsed six to eight times with ice‐cold distilled water, and 100 µl of destaining solution (75% ethanol) was added. Quantification of CV was performed by measuring the absorbance at 590 nm using a microplate reader.

### Cytotoxicity measurement

The toxicity of the compounds was assessed by measuring the release of lactate dehydrogenase (LDH) from human A549 cells (lung cancer cells). A549 cells in DMEM supplemented with 10% FBS were seeded in 96‐well tissue culture plates at 1.5 × 10^4^ cells/well one night in advance and incubated at 37°C with 5% CO_2_. To test the cytotoxicity of the compounds, the medium in the seeded plate was replaced by DMEM supplemented with 1% FBS and containing different concentrations of the tested compounds as indicated, the same volume of 0.2% DMSO as the control. To test the cytotoxicity of the compounds against *C. albicans*, the medium was replaced by *C. albicans* cells (OD_600_ = 0.1) treated with different compounds at different concentrations in DMEM supplemented with 1% FBS, the same volume of 0.2% DMSO as the control. After 8 h of incubation, the LDH released was measured, and the cytotoxicity was calculated as the ratio of LDH from the treated cells to the difference between the LDH values of the chemically lysed cells (maximum) and those of the DMSO‐treated cells (control).

### Mouse systemic infection model and oral mucosal infection model

For animal experiments, the initial infectious inocula of *C. albicans* SC5314 were harvested by centrifugation and then washed three times using cold sterile PBS (pH = 7.4). Cells were diluted to an OD_600_ of 0.5 with PBS to prepare the inocula. In the mouse systemic infection model, fungal cell suspensions (OD_600_ = 0.5) were injected via the tail vein into 6‐ to 8‐week‐old male BALB/C mice (*n* = 8) at a final volume of 200 µl. After 4 h postinoculation with *C. albicans*, the mice in the treatment groups were injected 200 µl compounds at the concentration of 10 µM, and the mice in the positive control group were injected with 200 µl fluconazole at the concentration of 10 µg ml^−1^, but the mice in the negative control group were injected with PBS contained the same volume 0.2% DMSO. Survival rates of mice were recorded daily for two weeks to determine the survival curves and were analysed using GraphPad Prism 8.

The protocol for the OPC model was based on a published study (Solis and Filler, 2012). Groups of mice (*n* = 3) were immunosuppressed with cortisone acetate at a concentration of 225 mg kg^−1^ using subcutaneous injections 1 day before inoculation. *C. albicans* SC5314 cells were collected by centrifugation, washed twice with PBS and then dissolved in PBS to obtain a suspension of 3 × 10^6^ cells ml^−1^. The calcium alginate swab was saturated with cold *C. albicans* suspension for at least 15 min. Before inoculation, the mice were anesthetized with 40 mg kg pentobarbital sodium (Yuanye Biotech, Shanghai, China) and then placed on prepared isothermal mats at 37°C and inoculated sublingually with the soaked swabs for 75 min. Two days later, 400 µl compounds at 10 µM or the same volume of PBS were given to the mice by intragastric once every day for a total of three times. Five days later, the mice were euthanized, and their tongues were bisected longitudinally. One half of each tongue was used for CFU analysis, and the other was processed for tissue histology.

### RNA extraction and quantitative real‐time PCR

RNA extraction and real‐time PCR assays were performed as described previously (Li *et al*., 2013, Li *et al*., 2014). Briefly, *C. albicans* SC5314 cells were inoculated into YPD medium and diluted to an OD_600_ of 0.1 in the absence and presence of hypocrellins A, B and C at a final concentration of 10 µM, the same volume of 0.2% DMSO as the control. Cells were collected by centrifugation and then washed with PBS after incubation for 6 h at 37°C. Total RNA was extracted using TRIzol reagent (Invitrogen, Carlsbad, California, USA) and quantified. cDNA was obtained via a reverse transcription reaction using the Reverse Transcription Kit (TaKaRa Biotechnology) followed by amplification with SYBR Green Real‐Time PCR Master Mix (Vazyme Biotech) with primers as shown in Table S2. Quantitative real‐time PCR was performed with a 7300Plus real‐time PCR system (Applied Biosystems, USA). The data were normalized to the expression level of the GSP1 gene. The relative fold changes in the target genes were calculated using the quantitative comparative CT(2^−ΔΔ^ *^CT^*) method.

### Statistics

All data were presented as the mean ± standard deviations from three independent experiments. All results were calculated from the means of three separate experiments. Statistical analysis was performed using GraphPad Prism 8 with the unpaired *t*‐test at a *P* < 0.05, *P* < 0.01 or *P* < 0.001 level of significance.

## Conflict of interests

The authors declare no conflicts of interest.

## Author contribution

Y. D. and S. S. designed the research; S. S. and X. S. performed the research; S. S., X. S., L. M., Q. W., K.W. and Y. D. analysed the data; and S. S. and Y. D. wrote the paper.

## Supporting information


**Table S1** **.** Summary of the compounds.
**Table S2** **.** Quantitative real‐time PCR primers used in this study.
**Fig. S1** **.** Analysis of the toxic effect of hypocrellins on *C. albicans* cells. (A) Effects of hypocrellins A, B and C on the growth rate of *C. albicans* SC5314 at a final concentration of 10 µM. (B) Analysis of the viability of *C. albicans* cells by Evan’s blue staining in the absence and presence of hypocrellins. (C) Microscopic observation of *C. albicans* cells in the absence and presence of hypocrellins by Evan’s blue staining. The cells stained in blue color were dead. *P < 0.05; ***P < 0.001 vs DMSO (unpaired *t*‐test).
**Fig. S2** **.** Analysis of the cytotoxicity of hypocrellin compounds and their effects on *C. albicans* cytotoxicity by using MTT assay. (A) Analysis of the toxicity of the hypocrellin compounds against A549 cells. (B) Analysis of the effects of the hypocrellin compounds on the cytotoxicity of *C. albicans* against A549 cells. (C) Analysis of the toxicity of the hypocrellin compounds against HIEC cells. (D) Analysis of the effects of the hypocrellin compounds on the cytotoxicity of *C. albicans* against HIEC cells. Compounds were dissolved in DMSO, and the same amount of DMSO (0.2%) used as the solvent for the compounds was used as a control. Data are means ± standard deviations from three independent experiments. *P < 0.05; **P < 0.01; ***P < 0.001 vs DMSO (unpaired *t*‐test).
**Fig. S3** **.** Effects of hypocrellins A, B and C (10 μM) on the growth rate of *C. albicans* ATCC 90028 (A), *C. glabrata* ATCC 2001 (B), *C. albicans* ATCC 14053 (C), *C. albicans* ATCC 10231 (D), and *C. tropicalis* ATCC 750 (E).
