# Supplementary material for: Antifungal activity of hypocrellin compounds and their synergistic effects with antimicrobial agents against Candida albicans
Source: Microb Biotechnol. 2020 Jun 8;14(2):430–43. doi: 10.1111/1751-7915.13601 (PMC7936304; doi:10.1111/1751-7915.13601)
Supplement: Supplementary file 1 — Table S1 . Summary of the compounds. Table S2 . Quantitative real‐time PCR primers used in this study. Fig. S1 . Analysis of the toxic effect of hypocrellins on C. albicans cells. (A) Effects of hypocrellins A, B and C on the growth rate of C. albicans SC5314 at a final concentration of 10 µM. (B) Analysis of the viability of C. albicans cells by Evan’s blue staining in the absence and presence of hypocrellins. (C) Microscopic observation of C. albicans cells in the absence and presence of hypocrellins by Evan’s blue staining. The cells stained in blue color were dead. *P < 0.05; ***P < 0.001 vs DMSO (unpaired t‐test). Fig. S2 . Analysis of the cytotoxicity of hypocrellin compounds and their effects on C. albicans cytotoxicity by using MTT assay. (A) Analysis of the toxicity of the hypocrellin compounds against A549 cells. (B) Analysis of the effects of the hypocrellin compounds on the cytotoxicity of C. albicans against A549 cells. (C) Analysis of the toxicity of the hypocrellin compounds against HIEC cells. (D) Analysis of the effects of the hypocrellin compounds on the cytotoxicity of C. albicans against HIEC cells. Compounds were dissolved in DMSO, and the same amount of DMSO (0.2%) used as the solvent for the compounds was used as a control. Data are means ± standard deviations from three independent experiments. *P < 0.05; **P < 0.01; ***P < 0.001 vs DMSO (unpaired t‐test). Fig. S3 . Effects of hypocrellins A, B and C (10 μM) on the growth rate of C. albicans ATCC 90028 (A), C. glabrata ATCC 2001 (B), C. albicans ATCC 14053 (C), C. albicans ATCC 10231 (D), and C. tropicalis ATCC 750 (E). [file MBT2-14-430-s001.pdf]

Supporting Information:

Antifungal activity of hypocrellin compounds and their synergistic effects with antimicrobial agents against *Candida albicans*

Shihao Song[1,2,†], Xiuyun Sun[2, †], Lili Meng[2], Qianhua Wu[2], Ke Wang[3], Yinyue Deng[1,2,*]

[1] *School of Pharmaceutical Sciences (Shenzhen), Sun Yat-sen University, Guangzhou 510275, China*

[2] *College of Agriculture, South China Agricultural University, Guangzhou 510642, China*

[3] *Pulmonary and Critical Care Medicine Ward, The First Affiliated Hospital of Guangxi Medical University, Nanning 530021, China*

[†]These authors have contributed equally to this work.

[*]Corresponding author:

Yinyue Deng: yinyue_deng@hotmail.com

Tel.: (+86)2085286471; Fax: (+86)2085280203

SI Methods

Candida cell growth analysis.

Candida cells cultured overnight were diluted in YPD medium and inoculated into fresh medium to an OD_{600} of 0.05 with or without compounds at the indicated final concentrations. Then, 200 µl of inoculated culture was grown in each well at 30°C under low-intensity shaking using the Bioscreen-C Automated Growth Curves Analysis System (OY Growth Curves AB, Finland).

Viability assay of *Candida* cells

The viability of cells was tested using 1% Evan's Blue dye, which is excluded by viable cells (1). Briefly, *candida* cells cultured overnight were diluted in YPD medium and inoculated into fresh medium to an OD_{600} of 0.5 with or without compounds at the indicated final concentrations of 10 µM. The inoculated cultures were grown in each well at 30°C as indicated with shaking at 220 rpm for 4 h. Then the same volume of 0.5% Evan's blue solution was added, and the cells were dyed at room temperature for 10 min. Finally, the samples were measured for the viability by an optical microscope.

MTT assay

MTT (3-[4,5-dimethylthiazol-2-yl]-2,5 diphenyl tetrazolium bromide) assay (MTT Cell Proliferation Kit, Sangon Biotech) is based on the conversion of MTT into formazan crystals by human cells, which is used to determine mitochondrial activity (2). A549 cells (lung cancer cells) and human intestinal epithelial cells (HIEC) in DMEM supplemented with 10% FBS were seeded in 96-well tissue culture plates at 1.5×10^4 cells/well one night in advance and incubated at 37°C with 5% CO_2. To test the cytotoxicity of the compounds, the medium in the seeded plate was replaced by 100 µL DMEM supplemented with 1% FBS and containing different concentrations of the tested compounds as indicated, the same volume of 0.2% DMSO was used as the control. To test the effects of the compounds against *C. albicans* cytotoxicity, the medium was replaced by *C. albicans* cells (OD_{600}=0.1) treated with different

compounds at different concentrations in DMEM supplemented with 1% FBS, the same volume of 0.2% DMSO was used as the control. 10 µL of 5 mg/mL MTT stock solution was added to each well and mixed gently and incubate for 4 hours. Then the culture medium was aspirated from each well carefully to prevent disruption of the cell monolayer. And 100 µL of formazan solubilization solution was added into each well and then the plates were placed on a shaker to mix gently for 10 minutes to dissolve the formazan crystals. The cytotoxicity was determined by measuring the absorbance at 570 nm using a microplate reader.

Analysis of *C. albicans* cell numbers in mouse tongue

The mouse tongue was collected and weighed, then ground in sterilized water. The mouse tongue suspension was serially diluted and spread on SD plates. The plates were grown at 30°C for two days. Then the numbers of *C. albicans* colony-forming unit (CFU) on the plates were counted.

Histopathological diagnosis

In order to study the oral cavity of mice infected with *C. albicans*, the tongues of mice were observed by pathological section. The histological sections were stained with Periodic Acid-Schiff stain for histopathological diagnosis (3). The mouse tongues were fixed with 4% formaldehyde, then dehydrated and paraffin embedded. The sections were dewaxed to water and stained with Periodic Acid-Schiff stain for histopathological diagnosis. Finally, they were observed and imaged with a microscope.

Analysis of susceptibility of *Candida* strains to compounds or antifungal agents

MIC values of antifungal agents and hypocrellins against *Candida* spp. used in this study were determined based on the broth microdilution protocol of Clinical and Laboratory Standards Institute (CLSI) document M23-A7 with small modifications (4, 5). Briefly, overnight cultured cells were inoculated at an OD_{600} of 0.05 in YPD medium supplemented with compounds or antifungal agents. One hundred microliters of

inoculated cultures were grown in each well at 30°C as indicated with shaking at 250 rpm for 24 hours. MIC was defined as the lowest concentration of antibiotic in which *Candida* spp. growth in the well was not measureable by determination of the turbidity at 600 nm following the method from the Clinical and Laboratory Standards Institute (CLSI).

Protein extraction and quantification

Candida cells cultured overnight were diluted in YPD medium and inoculated into fresh medium to an OD_{600} of 0.5 with or without compounds at the indicated final concentrations of 10 μM. The inoculated cultures were grown in each well at 37°C as indicated with shaking at 220 rpm for 8 h. *C. albicans* cells were harvested and washed twice with ice-cold PBS. The pellets were suspended in the residual PBS and transferred into 1.5 mL screw-cap tubes. After centrifugation for 2 min at 13,000×g, the pellets were suspended in 200 μL radio immunoprecipitation assay buffer (RIPA buffer) (Sigma-Aldrich Co., St. Louis, MO, USA). Glass beads (approx. 0.25 g) were added and cells were disrupted by grinding using a Bioprep-24 homogenizer (Allsheng Instruments CO., Ltd., Hangzhou, China) pre-chilled with ice for 5 min. Cell debris and glass beads were removed by centrifugation at 13,000 rpm for 10 min at 4 °C, and the resulting supernatants were stored at −20 °C (short-term) or at −80 °C (long-term). Protein concentration was determined by the Bio-Rad protein assay (Bio-Rad, Hercules, CA, USA).

Western blotting

Expression levels of EFG1, HWP1 and TEC1 were determined by Western blotting (6). Equal amounts of boiled protein were separated on a 10% SDS-PAGE and samples were transferred from PAGE gels onto polyvinylidene fluoride (PVDF) membranes (Millipore, Billerica, MA, USA). Membranes were blocked in TBS-T-M (0.1 M Tris-HCl, pH 7.4, 0.9% NaCl, 0.1% Tween-20, 5% skimmed dried milk) either at room temperature for 1h or at 4 °C overnight. After being washed twice with TBS-T (TBS containing 0.1% Tween20), the membranes were incubated with 15 mL of a 1:3,000

dilution (in TBS-T-M) of the primary rabbit polyclonal antibody (Dia-an Biotechnology Co., Ltd., Wuhan, China) at room temperature for 1 h. Membranes were then rinsed twice with PBS-T and washed with TBS-T once for 15 minutes and then three more times for 5 min. Incubation with the secondary antibody, a peroxidase-conjugated goat anti-rabbit IgG (Thermo Fisher Scientific) was carried out in the same way as the primary antibody. Membranes were rinsed and washed following the protocols as above, allowing the antibody-bound proteins to be detected by enhanced chemiluminescence (ECL; Thermo Fisher Scientific, Waltham, MA, USA), which was performed according to the manufacturer's instructions and was observed via Tanon 5200 (Tanon Science & Technology Co., Ltd., Shanghai, China).

Table S1. Summary of the compounds

Compound Number	Name	Structure	CAS Number	Hyphae Formation
1	Alkannin	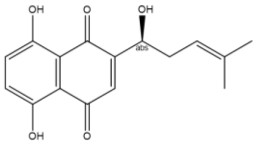	517-88-4	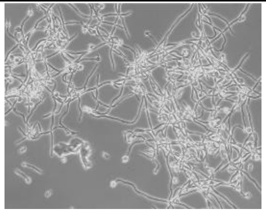
2	Aloe-emodin-8-O-beta-D-glucopyranoside	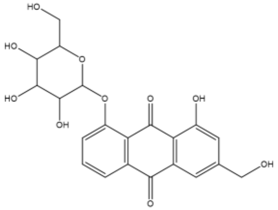	33037-46-6	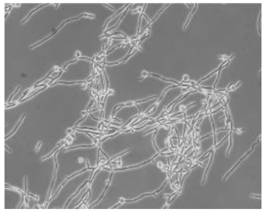
3	Aloenin A	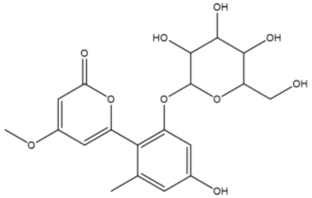	38412-46-3	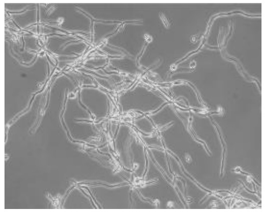
4	Hypocrellin A	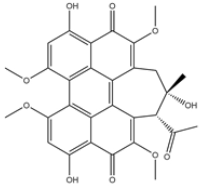	77029-83-5	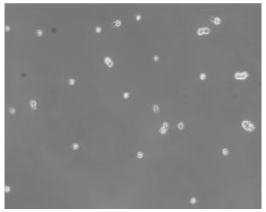

5	Hypocrellin B	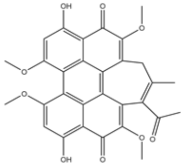	123940-54-5	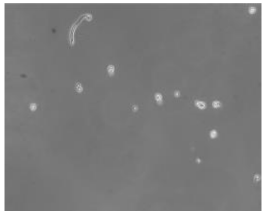
6	Hypocrellin C	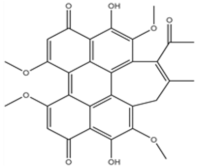	149457-83-0	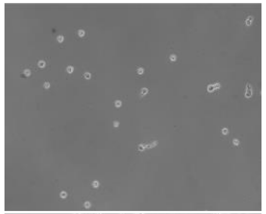
7	1,2,3,4,6-Pentagalloylglucose	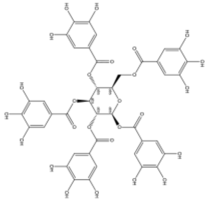	14937-32-7	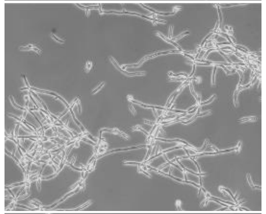
8	1,3,6-Tri-O-galloyl-beta-D-glucose	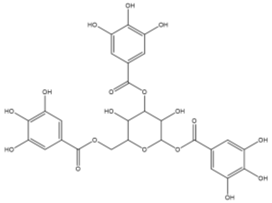	18483-17-5	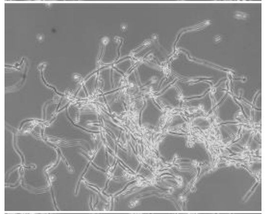
9	Chrysophanol-8-O-beta-D-glucopyranoside	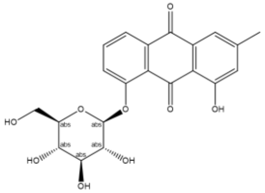	13241-28-6	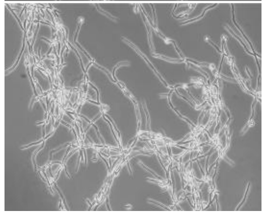

10	Emodin	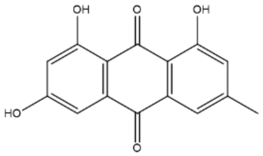	518-82-1	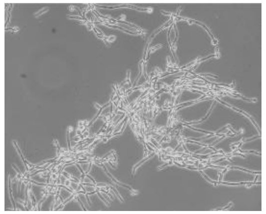
11	Emodin 1-glucoside	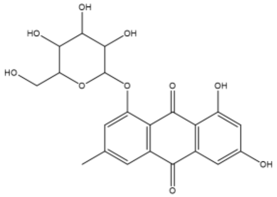	38840-23-2	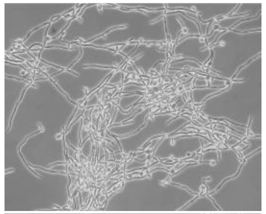
12	Emodin-8-glucoside	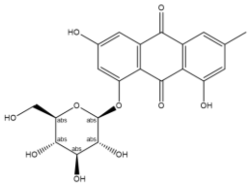	23313-21-5	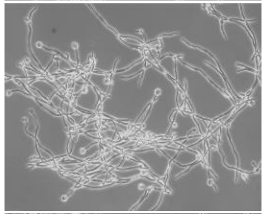
13	Hypericin	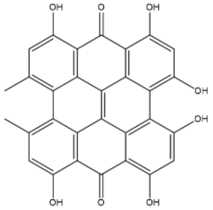	548-04-9	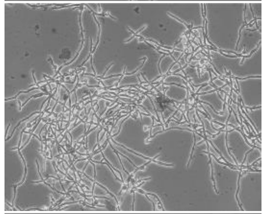
14	Juglone	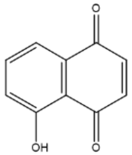	481-39-0	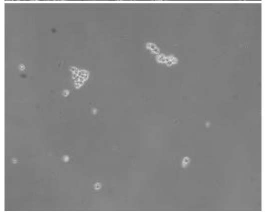

15	plumbapin	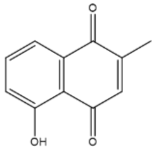	481-42-5	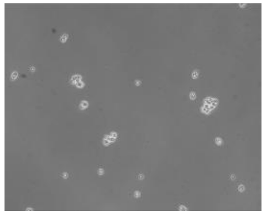
16	Sennoside A	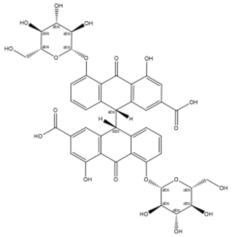	81-27-6	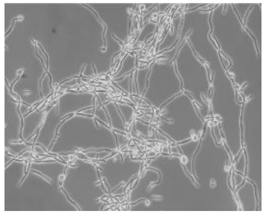
17	Sennoside B	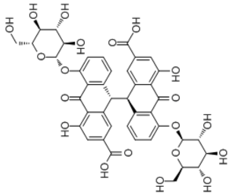	128-57-4	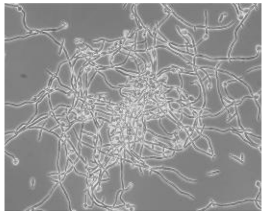
18	Sennoside C	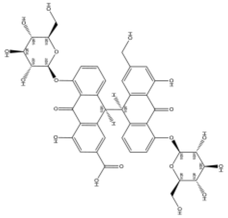	37271-16-2	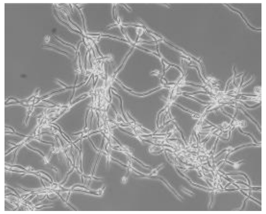

19	Sennoside D	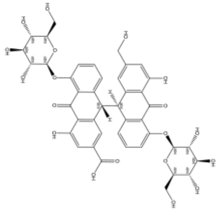	37271-17-3	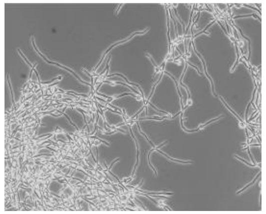
20	Rhein-8-glucoside	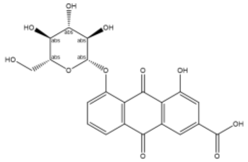	34298-86-7	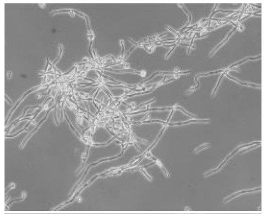
21	Tinnevellin glucoside	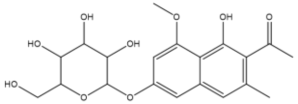	80358-06-1	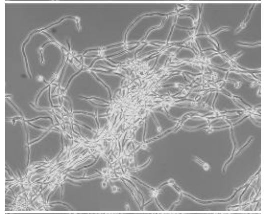
22	Protohypericin	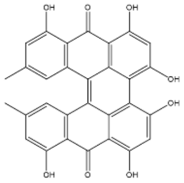	548-03-8	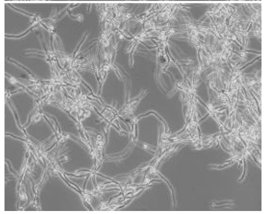
23	Diacerein	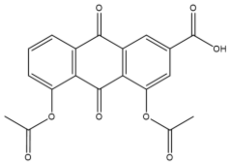	13739-02-1	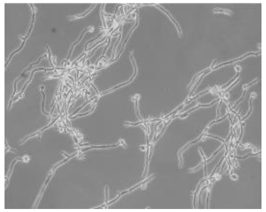

24	Beta-Hydroxylsovalerylshikonin	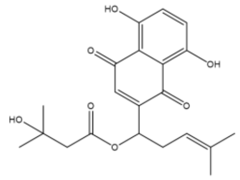	7415-78-3	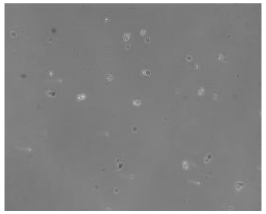
25	Aurantio-obtusin β-D-glucoside	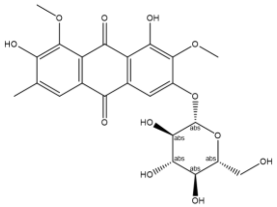	129025-96-3	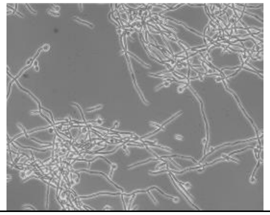

C. albicans SC5314 cells were induced at 37°C for 6 h. The concentration of compounds is 10 μM. Images of cells were captured by a Leica inverted fluorescence microscope with 40× lenses.

Table S2. Quantitative real-time PCR primers used in this study

Primer	Sequence (5'-3')
GSP1-F[a]	TGAAGTCCATCCATTAGGAT
GSP1-R	ATCTCTATGCCAGTTTGGAA
HWP1-F	TGGTGCTATTACTATTCCGG
HWP1-R	CAATAATAGCAGCACCGAAG
EFG1-F	TATGCCCCAGCAAACAACTG
EFG1-R	TTGTTGTCCTGCTGTCTGTC
ALS3-F	CTAATGCTGCTACGTATAATT
ALS3-R	CCTGAAATTGACATGTAGCA
CST20-F	TTCTGACTTCAAAGACATCAT
CST20-R	AATGTATATTTCTGGTGGTG
CDC35-F	TTCATCAGGGGTTATTTCAC
CDC35-R	CTCTATCAACCCGCCATTTC
TEC1-F	AGGTTCCCTGGTTTAAGTG
TEC1-R	ACTGGTATGTGTGGGTGAT
CPH1-F	ATGCAACACTATTTATACCTC
CPH1-R	CGGATATTGTTGATGATGATA
ECE1-F	GCTGGTATCATTGCTGATAT
ECE1-R	TTCGATGGATTGTTGAACAC
PDE2-F	ACCACCACCACTACTACTAC
PDE2-R	AAAATGAGTTGTTCCTGTCC
HST7-F	ACTCCAACATCCAATATAACA
HST7-R	TTGATTGACGTTCAATGAAGA

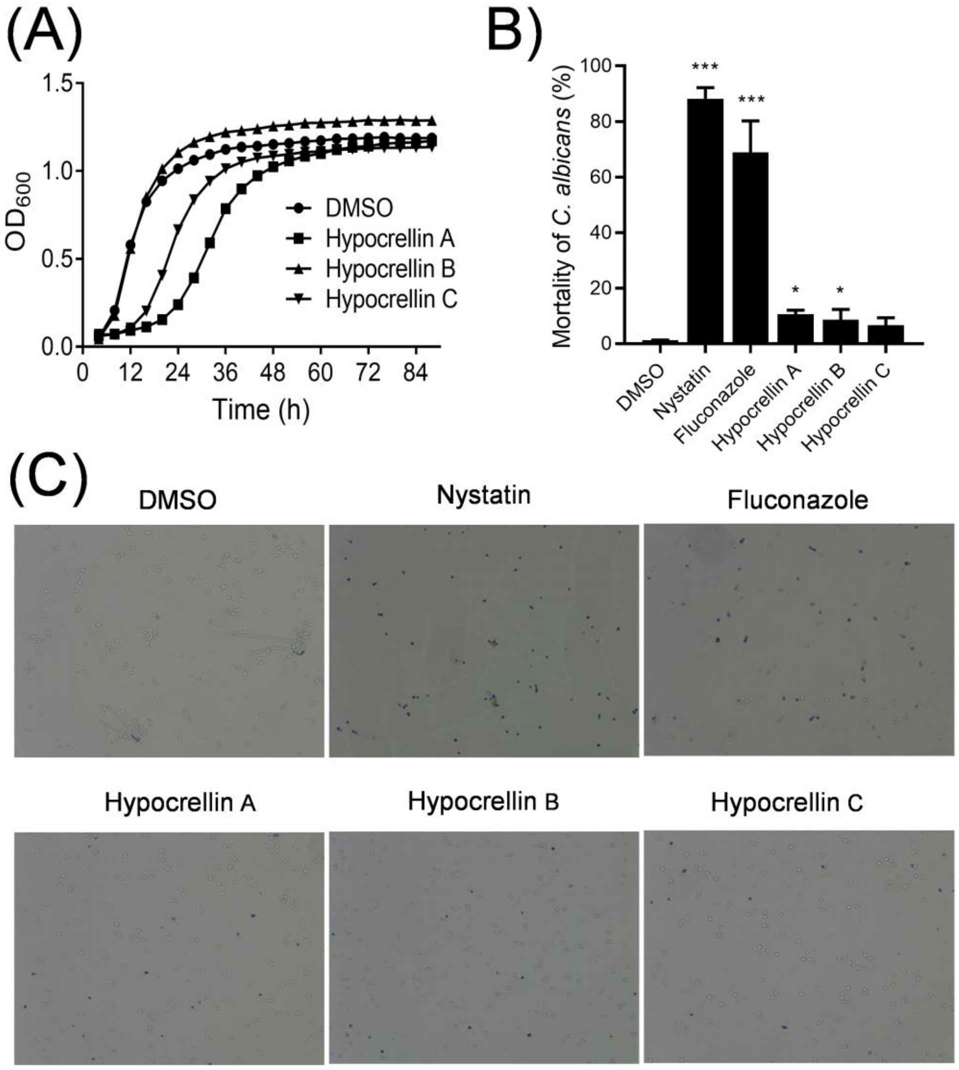

Fig. S1. Analysis of the toxic effect of hypocrellins on *C. albicans* cells. (A) Effects of hypocrellins A, B and C on the growth rate of *C. albicans* SC5314 at a final concentration of 10 μM. (B) Analysis of the viability of *C. albicans* cells by Evan's blue staining in the absence and presence of hypocrellins. (C) Microscopic observation of *C. albicans* cells in the absence and presence of hypocrellins by Evan's blue staining. The cells stained in blue color were dead. $*P < 0.05$; $***P < 0.001$ vs DMSO (unpaired t-test).

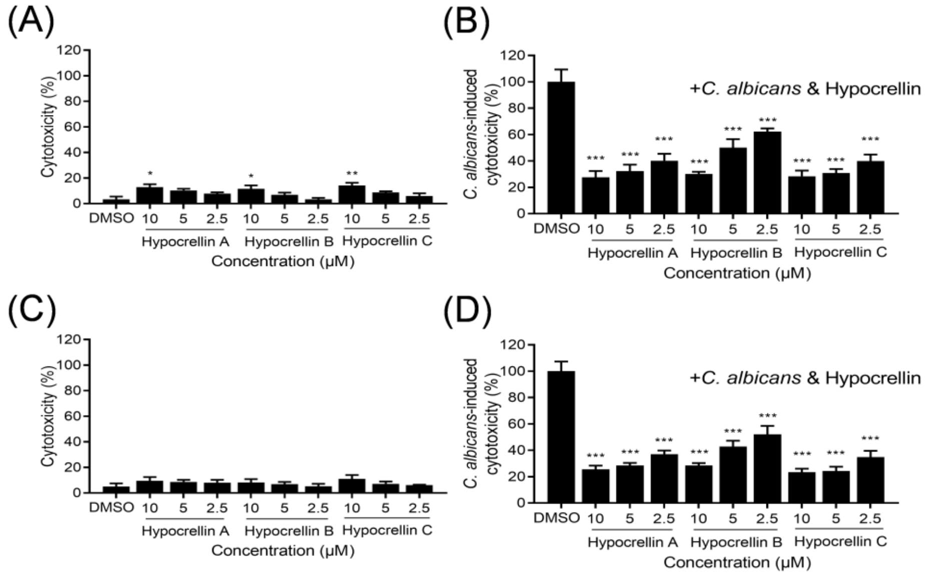

Fig. S2. Analysis of the cytotoxicity of hypocrellin compounds and their effects on *C. albicans* cytotoxicity by using MTT assay. (A) Analysis of the toxicity of the hypocrellin compounds against A549 cells. (B) Analysis of the effects of the hypocrellin compounds on the cytotoxicity of *C. albicans* against A549 cells. (C) Analysis of the toxicity of the hypocrellin compounds against HIEC cells. (D) Analysis of the effects of the hypocrellin compounds on the cytotoxicity of *C. albicans* against HIEC cells. Compounds were dissolved in DMSO, and the same amount of DMSO (0.2%) used as the solvent for the compounds was used as a control. Data are means ± standard deviations from three independent experiments. *$P < 0.05$; **$P < 0.01$; ***$P < 0.001$ vs DMSO (unpaired t-test).

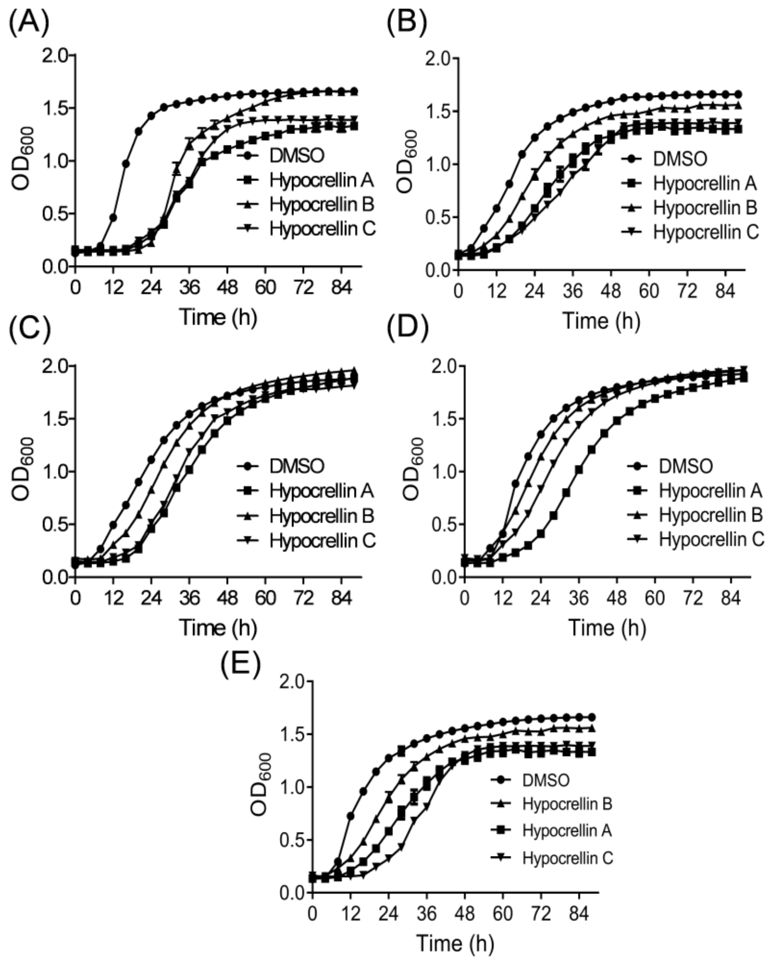

Fig. S3. Effects of hypocrellins A, B and C (10 μM) on the growth rate of *C. albicans* ATCC 90028 (A), *C. glabrata* ATCC 2001 (B), *C. albicans* ATCC 14053 (C), *C. albicans* ATCC 10231 (D), and *C. tropicalis* ATCC 750 (E).

References

1. Papazi, A., Makridis, P., and Divanach, P. (2010) Harvesting *Chlorella minutissima* using cell coagulants. *Journal of Applied Phycology*, **22**:349-355.
2. Meerloo, J.V., Kaspers, G.J.L., and Cloos, J. (2011). Cell sensitivity assays: The MTT assay. *Methods in molecular biology (Clifton, N.J.)*, **731**:237-245.
3. Chen, L., Wang, Z., Liu, L., Qu, S., Mao, Y., Peng, X., L *et al.* (2019) Cinnamaldehyde inhibits *Candida albicans* growth by causing apoptosis and its treatment on vulvovaginal candidiasis and oropharyngeal candidiasis. *Applied microbiology and biotechnology*, **103**:9037-9055.
4. Clinical Laboratory Standards Institute. (2008) Reference method for broth dilution antifungal susceptibility testing of yeasts. Approved standard M27-A3, 3rd ed. Clinical Laboratory Standards Institute, Wayne, PA.
5. Konaté, K., Mavoungou, J.F., Lepengué, A.N., Aworet-Samseny, R.R., Hilou, A., Souza, A., *et al.* (2012) Antibacterial activity against β-lactamase producing Methicillin and Ampicillin-resistant *Staphylococcus aureus*: fractional Inhibitory Concentration Index (FICI) determination. *Annals of Clinical Microbiology and Antimicrobials*, **11**:18.
6. Han, Q., Wang, N., Yao, G., Mu, C., and Sang, J. (2018). Blocking β-1,6-glucan synthesis by deleting KRE6 and SKN1 attenuates the virulence of *Candida albicans*. *Molecular Microbiology*, **111**:604-620.
